# Periodontal health in teeth treated with deep-margin-elevation and CAD/CAM partial lithium disilicate restorations—a prospective controlled trial

**DOI:** 10.1007/s00784-024-06053-y

**Published:** 2024-11-30

**Authors:** Tim Hausdörfer, Clemens Lechte, Philipp Kanzow, Tina Rödig, Annette Wiegand

**Affiliations:** Department of Preventive Dentistry, Periodontology and Cariology, University Medical Center Göttingen, Robert-Koch-Str. 40, 37075 Göttingen, Germany

**Keywords:** CAD/CAM partial-coverage crowns, Cervical margin relocation, Deep-margin-elevation, Periodontal health, Proximal box elevation

## Abstract

**Objectives:**

This prospective controlled clinical trial aimed to compare periodontal parameters of proximal deep-margin-elevation (DME) restoration margins with supragingival/equigingival restoration margins (control) on the opposite proximal surface of the same tooth.

**Materials and methods:**

Subgingival one-sided proximal defects (mesial or distal) on (pre-)molars were restored with composite DME and CAD/CAM-manufactured lithium disilicate ceramic partial-coverage restorations. Periodontal parameters (bleeding on probing (BOP), periodontal probing depths (PPD), plaque index (PI)) were recorded after insertion of the ceramic restoration (baseline) and at 1-year recall visit and compared between DME and control on the same tooth (Fisher’s exact test and Wilcoxon signed rank test, *p* < 0.05).

**Results:**

Sixty-eight patients with 77 restorations were included. At baseline, periodontal parameters did not differ between DME and control. Sixty-two restorations could be examined after 1 year. BOP was significantly increased for DME (p_adj._ = 0.003), but not for control (p_adj._ = 0.714). Surfaces with DME showed a significantly higher proportion of BOP than control surfaces (DME: 45 restorations (73.8%), control: 27 restorations (44.3%); p_adj._ = 0.005). PI increased significantly on all tooth surfaces (p_adj._<0.001), but did not differ between DME and control side (p_adj._ = 0.162). Probing depths did not differ between baseline and follow-up (DME: p_adj._ = 0.199, control: p_adj._ = 0.116). Two restorations were replaced due to a ceramic fracture and secondary caries.

**Conclusion:**

Proximal DME is associated with increased gingival inflammation compared to supragingival or equigingival restoration margins.

**Clinical relevance:**

DME is a promising treatment approach for indirect restoration of teeth with deep proximal defects, but gingival inflammation should be expected.

## Introduction

The treatment of deep subgingival defects is challenging due to difficult moisture and contamination control. Imprecisely processed restorations, small margin leaks, or protruding edges promote plaque accumulation and have a critical effect on the periodontal tissues [1, 2]. There is also evidence that subgingival restorations that violate the biological width cause gingival inflammation [3, 4]. The classic treatment option is surgical crown lengthening to protect the supra-crestal attachment. However, surgical crown lengthening has some disadvantages such as prolonged treatment time and costs, compromised dental aesthetics, loss of attachment, and proximity to root concavities and furcation areas [5]. Current trends in tooth preservation are to treat teeth as minimally invasive as possible and to avoid surgical procedures [6].

Deep-margin-elevation (DME) is a method that has been described for several years: Deep cavity margins are elevated to the level of the cementoenamel junction using direct composite restorations. This procedure simplifies digital scans or conventional impressions, the isolation with rubber dam, and cementation of ceramic restorations [7]. In-vitro studies on the marginal quality of ceramic restorations after proximal DME showed comparable results to restorations with a supragingival margin preparation [8, 9].

To date, there have only been a few clinical studies that examined teeth with DME [10], with follow-up periods varying from 3 months to 21 years [11, 12]. To restore teeth after DME, both direct composite restorations [13] and indirect inlays or partial-coverage crowns made of CAD/CAM-manufactured resin composite restorations or lithium disilicate ceramics were reported [12, 14–16]. With regard to survival and success rates, the available clinical studies consider DME as a promising and reliable treatment option [14, 15, 17].

The impact of DME on periodontal health has only been analyzed in a few studies [6, 11, 13, 15]. Ferrari et al. [15] showed increased bleeding on probing (BOP) if the distance between the restoration margin and the alveolar bone is less than 2 mm. In contrast, subgingival composite resin restorations with DME did not show increased signs of inflammation compared to other tooth surfaces during follow-up, provided that meticulous interdental cleaning with regular use of interdental brushes was performed [13]. This clinical observation is also underlined by histological studies showing that periodontal tissues around root surfaces with subgingival restoration margins are comparable to natural root surfaces [11]. A randomized clinical trial compared periodontal parameters of teeth with DME and surgical crown lengthening and reported no significant differences regarding inflammatory parameters. However, probing depths were higher in the surgical crown lengthening group after 9 and 12 months than in the DME group [6].

Regarding the current evidence, some authors recommend DME to be preferred over surgical crown lengthening due to its minimally invasive nature [17]. However, the effect of DME on periodontal tissue is still not fully understood [17].

Thus, this prospective controlled clinical trial aimed to compare the periodontal parameters and biological properties of proximal composite DME restoration margins with supragingival or equigingival restoration margins of a CAD/CAM partial lithium disilicate restoration on the opposite proximal surface of the same tooth. The null hypothesis was that DME does not impact periodontal parameters.

## Materials and methods

The study was approved by the local ethics committee of the University Medical Center Göttingen (approval no: 11/3/17) and registered in the German Clinical Trials Register (DRKS-ID: DRKS00016803) prior to its initiation.

### Sample size calculation

Sample size calculation was performed based on the results of the study by Ferrari et al. [15]. The 1-year control of teeth with a DME revealed a positive BOP in 53% of cases in the DME-group (compared to 0% at baseline) and 31.5% of cases in the control group (compared to 0% at baseline). The sample size planning was carried out using a chi-square test with α = 0.05 and β = 0.2 (G*Power, version 3.1.9.3) and resulted in *n* = 61. Since the recruitment period and the time of the follow-up examination fell within the restrictions on outpatient care caused by the COVID-19 pandemic, we assumed a dropout rate of 25%, which resulted in an anticipated number of 81 restorations at baseline.

### Patient population and recruitment

**Fig. 1:**
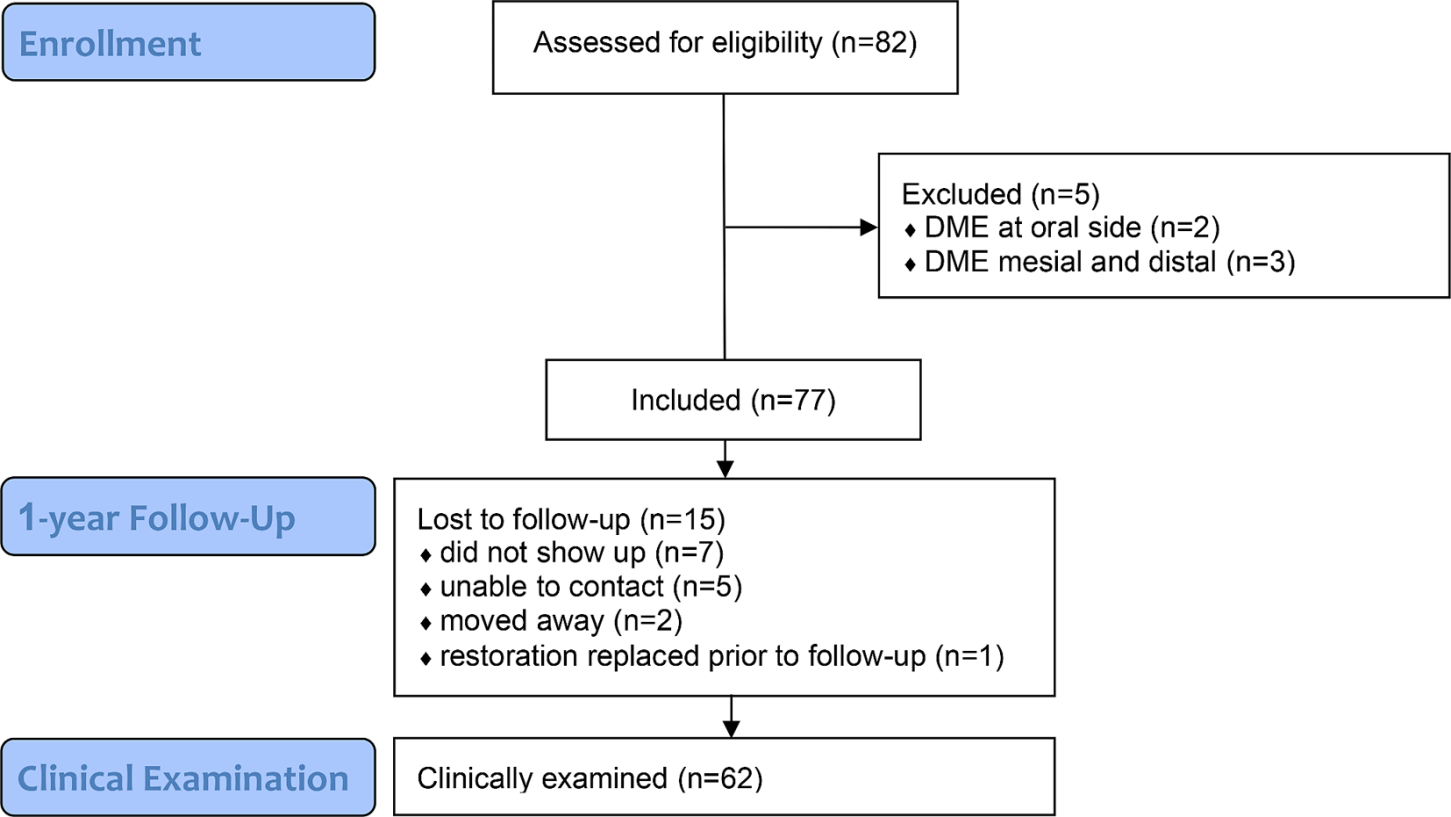
Study flow diagram. DME: deep-margin-elevation

The patients were recruited and treated in the Department of Preventive Dentistry, Periodontology and Cariology of the University Medical Center Göttingen between January 2019 and March 2023. Potentially eligible patients were identified during routine clinical care. Inclusion criteria were adult patients with an indication for a partial ceramic restoration and a subgingival defect in one proximal area on a vital or root canal treated premolar or molar. Only teeth with probing depths up to 5 mm and a tooth mobility score up to I were included. Teeth with apical periodontitis and teeth with missing antagonist dentition were excluded. Further exclusion criteria were pregnancy, breastfeeding, known allergies to materials used in the study, and severe systemic illnesses that hinder treatment. All teeth were treated as part of the routine dental therapy plan. An informed consent form was signed by all participants before the treatment. The study flow diagram is shown in Fig. 1.

### DME

The treatment was carried out by trained dentists or by students under their supervision. All patients received general oral hygiene instructions prior to therapy, including interdental cleaning with dental floss or an interdental brush. Vital teeth were anesthetized with Articain (Ultracain DS; Septodont, Niederkassel, Germany). If possible, rubber dam was used for isolation of the operating field. Pre-existing restorations were removed using a handpiece with diamond burs of different grit sizes, caries lesions were removed using round steel burs. Only if proximal subgingival defects could not be isolated with rubber dam, defects were raised to the level of the cementoenamel junction. Therefore, the rubber dam was cut in the respective proximal area, and DMEs were performed using metal matrices (Palodent Matrix Systems or AutoMatrix; Dentsply Sirona, Bensheim, Germany), wedges, and Teflon tape. DME was only carried out if the matrices were able to completely isolate the defect. Prior to the DME, a 3step adhesive in etch&rinse mode (Optibond FL; Kerr, Bioggio, Switzerland) was used. Enamel surfaces were etched with phosphoric acid (35% Ultra etch; Ultradent, South Jordan, UT, USA) for 30 s, and dentine surface were etched for 15 s. The primer was applied for 20 s, followed by the adhesive and light-activated polymerization (Curing light, BA optima, Northampton, UK) for 20 s. A flowable bulk fill composite (SDRflow; Dentsply Sirona) was used to elevate the subgingival defect up to the cementoenamel junction. After light curing of the composite for 40 s and the matrix and rubber dam were removed. Finally, excess adhesive and composite were removed using hand scalers (S204S6; Hu-Friedy, Chicago, IL, USA).

### Tooth preparation and CAD/CAM-workflow

All teeth were prepared for an multi-surface inlay or partial crown according to the guidelines for ceramic restorations [18]. Except for the area with the DME, all preparation margins were placed within the dental hard tissues. The clinical situation was digitally scanned using intraoral cameras (CEREC Primescan or CEREC Omnicam; Dentsply Sirona). The digital construction of the restoration was carried out using the CEREC system (CEREC Software 5.x; Dentsply Sirona). If a chairside fabrication was not possible due to time constraints, a temporary restoration (Luxatemp; DMG, Hamburg, Germany) was placed with a eugenol-free temporary cement (Temp Bond NE; Kerr).

For all teeth, lithium disilicate ceramic (IPS e.max CAD; Ivoclar Vivadent, Schaan, Lichtenstein) was used, which was crystallized and glazed (Vacumat 6000 M; VITA Zahnfabrick H. Rauter, Bad Säckingen, Germany) after the milling process (Cerec MC XL; Dentsply Sirona). Following trying-in, the restoration was cleaned, conditioned using hydrofluoric acid (Porcelain etch 9%; Ultradent), and silanized for 60 s (Monobond Plus; Ivoclar Vivadent). The prepared tooth and the neighboring teeth were cleaned and isolated with rubber dam (sigma dam; Sigma dental systems, Handewitt, Germany). Subsequently, the DME surface was silicated (CoJet sand; 3 M ESPE, St. Paul, MN, USA). Remnants of the blasting agent were removed using water. Selective enamel etching was performed for 30 s with phosphoric acid, and the surfaces were thoroughly rinsed with water. A universal primer (Monobond Plus) was applied on the surface of the DME for 60 s, followed by a universal adhesive (Adhese universal; Ivoclar Vivadent) which was light cured for 20 s. The restoration was luted using a flowable dual-curing composite (Variolink esthetik DC; Ivoclar Vivadent). Any excess material was removed with scalers and dental floss. The final light-activated polymerization time amounted to 40 s from each side. After removing the rubber dam, protruding restoration margins were trimmed, and the occlusion was adjusted with diamond burs. Finally, all surfaces were polished using silicone polishers.

### Evaluation

One of two calibrated examiners (T.R., A.W.) evaluated the restorations. Calibration was performed as part of a previous study on the clinical performance of CAD/CAM-manufactured partial crowns ([19], inter-rater reliability for periodontal parameters: 0.633). The examiners were blinded, as they were not involved in the treatment process and were not informed about the site of the DME. The clinical examination was performed at baseline (within a period of up to two weeks) and after one year. Periodontal assessment included pocket probing depths (mm) at 6 points of the tooth with a periodontal probe (XP3AUNC15; Hu-Friedy). In addition, BOP and the plaque index (PI) according to Silness and Löe [20] were recorded on both sides (i.e. sides with and without DME). The BOP was assessed as positive for the respective side if bleeding occurred at least at one probing site per proximal area. The highest plaque amount per proximal side determined the PI score (scores 0–4).

The restorations were assessed according to the World Dental Federation (FDI) criteria [21]. The selected functional FDI criteria were fracture of material and retention, marginal adaptation, proximal contact point, assessment of restoration on radiographs (if available), and patient’s view. Biological criteria were postoperative hypersensitivity and pulpal status (cold stimulation), caries, erosion, abfraction at restoration margins, integrity, and fracture of the tooth. The criteria were evaluated using the following scores: 1 clinically excellent/very good (sufficient); 2 clinically good (sufficient); 3 clinically satisfactory/acceptable (sufficient); 4 clinically unsatisfactory (partially insufficient); 5 clinically poor (entirely insufficient). Restorations rated with scores 1–3 were considered as success, i.e. the restoration had an intervention-free functional period until examination. Restorations rated with scores 1–4 were considered as survival, i.e. the restorations were in situ at the time of the examination. For ethical reasons, radiographs were only taken when clinically indicated and not on study purposes.

### Statistical analysis

Statistical analysis was performed using the software R (www.r-project.org, version 4.4.1). The assessed parameters were compared between sides with and without PBE (control) using Fisher’s exact test (BOP) and Wilcoxon signed rank test (PI, PPD). Also, comparison between values at baseline and at the 1-year recall was performed using Fisher’s exact test (BOP) and Wilcoxon signed rank tests (PI, PPD). P-values were adjusted for multiple testing according to Bonferroni-Holm (p_adj._ < 0.05).

## Results

Eighty-two restorations were assessed for eligibility, of which 5 were excluded because the DME was located on the oral tooth surface (*n* = 2) or was found on both proximal areas (*n* = 3). Finally, a total of 77 restorations placed in 68 patients (mean age: 42.7 ± 14.3 years, *n* = 35 female, *n* = 33 male) were included in the study. Twenty-nine teeth (37.7%) were vital and 48 (62.3%) underwent previous root canal treatment. The restorations were made by experienced dentists (*n* = 23, 29.9%) or by students under supervision of experienced dentists (*n* = 54, 70.1%). DME was performed in 49 distal (62.3%) and 29 (37.7%) mesial cavities. Most restorations had two proximal contacts (*n* = 63), while *n* = 14 teeth presented one proximal contact. After the 1-year recall interval (min: 0.9, max. 1.5 years), 62 restorations placed in 55 patients (*n* = 28 female, *n* = 27 male) could be clinically examined. The delayed recall interval (beyond 12 months) was due to the restrictions on patient care during the COVID-19 pandemic. One restoration was replaced before the end of the 1-year interval because of an irreparable ceramic fracture. The remaining 14 restorations could not be examined due to loss of follow-up (Fig. 1).

**Fig. 2:**
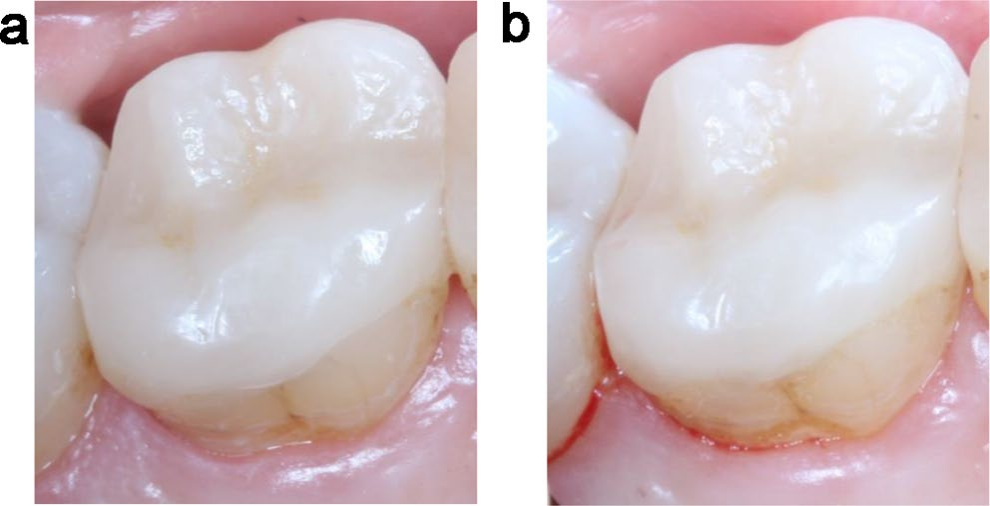
Papillae bleeding after measurment of periodontal probing dephts (**a**: baseline, **b**: 1-year recall visit)

Periodontal parameters are presented in Table 1. At baseline, all periodontal parameters did not significantly differ between DME and control. At the follow-up, the proportion of positive BOP was significantly increased for DME (p_adj._ = 0.003), but not for the control (p_adj._ = 0.714) compared to baseline (Fig. 2). Moreover, at the 1-year recall, the BOP was significantly higher in the proximal areas with DME than in control margins (p_adj._ = 0.005). From the baseline examination to the 1-year recall, the PI increased significantly on all tooth surfaces (p_adj._ < 0.001), but did not differ between the DME and the control side (p_adj._ = 0.162). The probing depths on all tooth surfaces did not differ between baseline examination and follow-up (p_adj._ = 0.199 for DME, p_adj._ = 0.116 for control), and there was no difference between the proximal areas with and without DME (p_adj._ = 0.162).

**Table 1:** Periodontal parameters in DME and control groups at baseline and the 1-year recall

Parameter	Baseline			1-year recall		
	DME	control	*p*_adj_.	DME	control	*p*_adj_.
BOP+, n (%)	32 (41.6)	32 (41.6)	> 0.999	45 (73.8)	27 (44.3)	**0.005**
PI, mean ± SD	0.13 ± 0.38	0.13 ± 0.38	> 0.999	1.26 ± 0.97	1.03 ± 0.92	0.162
PPD [mm], mean ± SD	2.57 ± 0.75	2.43 ± 0.69	0.094	2.72 ± 0.93	2.58 ± 0.69	0.162

Significant p-values are printed in bold. BOP+: positive bleeding on probing; DME: side with deep-margin-elevation; control: side with supragingival or equigingival restoration margin; PI: plaque index; PPD: periodontal probing depths in mm; SD: standard deviation

Two restorations failed during the observation period (fracture *n* = 1, secondary caries *n* = 1) and were rated with FDI score 5. In three teeth, there was a loss of retention of the ceramic restorations. These restorations could be reluted and were rated with FDI score 4.

## Discussion

In this study, gingival inflammation at the restoration margins with DME was significantly increased compared to supra-/equigingival restoration margins after 12 months. The BOP increased significantly for DME but not for control sides and, thus, was significantly higher for DME compared to supra- or equigingival restoration margins at the 1-year recall. This result is in line with the study by Ferrari et al. [15], which investigated the effects of subgingival composite DME on periodontal health in teeth restored with partial lithium disilicate restorations and showed a significantly increased BOP in the DME-group compared to the control-group at the 12-month follow-up.

Potential reasons for increased gingival inflammation in the DME group are irregularities in the margin design, e.g. overhanging restoration margins [2, 22] and margins placed within the area of the biological width [23]. In contrast to the present study, similar trials did not detect changes in BOP after an observation period of up to three years [6, 13]. This finding may be related to strict exclusion of patients with poor oral hygiene. Furthermore, in a study by Muscholl et al. [13], the low prevalence of gingival inflammation was attributed to a better margin quality resulting from the applied “snowplow technique” and to the individual instructions for interdental care with special brushes adapted to the DME area. In the present study, inadequate oral hygiene was not an exclusion criterion. All patients received general oral hygiene instructions including information on interdental care before treatment. However, no individual adjustments of interdental brushes were made.

At the 1-year-recall, no significant changes in probing depths could be detected, which was also shown in previous studies [6, 11, 15]. Currently, there is no evidence that DME has a negative influence on the histological and clinical structures of the periodontal attachment, if the biological width is not exceeded [11]. However, the violation of the biological width by the DME could lead to increased gingival inflammation and increased probing depths [23]. It is assumed that the study period was too short to show an effect on the probing depths; further clinical follow-ups are necessary.

Over the study period PI increased significantly on both sides but did not differ between DME and control. This also corresponds to observations from other clinical studies [15, 24]. In general, restorations do not exhibit perfect marginal adaptation [25], which promotes plaque accumulation on subgingival and supragingival restorations. The reasons for imperfect marginal quality might be associated with the preparation design [26] and the CAD/CAM-workflow, e.g. when determining the preparation margin or in the milling process [27]. In addition, the margin quality of adhesively bonded restorations decreases over time under thermomechanical loading, although there is no difference between the surfaces with and without DME [28].

During the study period, two restorations had to be removed and replaced. CAD/CAM-manufactured adhesively bonded partial-coverage crowns made from lithium disilicate ceramics without DME have a cumulative survival rate of 94.6% and a cumulative success rate of 91.6% after 2 years [19]. Due to the short observation period, final conclusions regarding the impact of DME on long-term survival or success rates of ceramic restorations cannot be drawn.

This prospective observational study evaluated the influence of DME on periodontal health by using the supra-/equigingival proximal restoration margins on the same tooth as a control group. Alternatively, DME could have been compared to sound teeth or surgical crown lengthening, as done in previous studies [6, 13]. Surgical crown lengthening allows for accessing deep subgingival preparation margins beyond the capacity of DME by placing the supporting tissues in a more apical region. Periodontal health in DME and surgical crown lengthening was compared by Farouk et al. [6], which found no significant difference at the 12-month-recall with regard to the BOP, but a significantly lower PDD and clinical attachment loss for DME. These findings were attributed to differences in the postsurgical remodelling processes.

The strengths of the present study include the prospective study design, which allows not only for comparison of DME and non-DME sides but also for the detection of differences over time, which will become more relevant in further follow-ups. Moreover, compared to previous prospective studies [6, 15], a larger sample size was achieved. The patient group selected was quite heterogeneous in terms of tooth location, pulp vitality, previous root canal treatment, age, sex, and overall oral health. This ensured that this study was not limited to experienced practitioners and/or a highly selective patient cohort [29]. As a limitation, different experienced and less-experienced operators performed the treatment. However, a recent study found no impact on clinical performance of CAD/CAM-restorations between experienced and less-experienced operators [19]. A further limitation is the limited observation period of only one year and the moderate inter-rater reliability. However, periodontal health will be further monitored over time.

The DME technique is useful for restoring subgingival defects, as it simplifies procedures such as intraoral scanning and rubber dam isolation for adhesively bonded ceramic restorations. However, this study found that DME-treated tooth surfaces are associated with increased gingival inflammation, highlighting the challenges in treating subgingival defects and maintaining proper oral hygiene. Further long-term clinical studies are needed to assess the effects of DME on periodontal tissue, particularly attachment loss, and on the survival rates of CAD/CAM restorations.

## Conclusion

With the limitations of this study and the short follow-up period of one year, restoration margins with DME present gingival inflammation to a larger extent than supra-/equigingival restoration margins. Longer observation times are necessary to determine whether the gingival inflammation results in a loss of periodontal attachment.

## Data Availability

No datasets were generated or analysed during the current study.
